# Personalized Anticoagulation in Cancer Patients: Current Evidence and Future Perspectives

**DOI:** 10.3390/medicina62091677

**Published:** 2026-08-31

**Authors:** Ștefan Chiorescu, Mihaela Mocan, Bianca Patricia Dinică, Ovidiu Nicolae Grad, Vlad-Ionuț Nechita, Roxana Mihaela Chiorescu

**Affiliations:** 1 Department of Surgery, Iuliu Hațieganu University of Medicine and Pharmacy, 400012 Cluj-Napoca, Romania; stefanc74@yahoo.com (Ș.C.); ovidiugrad@gmail.com (O.N.G.); 2 Department of Surgery, Emergency Clinical County Hospital, 400006 Cluj-Napoca, Romania; 3 Internal Medicine Department, Iuliu Hațieganu University of Medicine and Pharmacy, 400012 Cluj-Napoca, Romania; mihaela.mocan@gmail.com (M.M.); roxana.chiorescu11@gmail.com (R.M.C.); 4 Department of Internal Medicine, Emergency Clinical County Hospital, 400006 Cluj-Napoca, Romania; 5 “Prof. Dr. Octavian Fodor” Regional Institute of Gastroenterology and Hepatology, 400162 Cluj-Napoca, Romania; 6 Department of Surgery, Dr. Constantin Papilian Military Emergency Clinical Hospital, 400394 Cluj-Napoca, Romania; 7 Department of Fundamental Sciences, Discipline of Medical Informatics, Research Methodology and Data Analysis, Faculty of Nursing and Health Sciences (FAMSS), Iuliu Hațieganu University of Medicine and Pharmacy, 400012 Cluj-Napoca, Romania; nechita.vlad@umfcluj.ro

**Keywords:** cancer-associated thrombosis, venous thromboembolism, direct oral anticoagulants, low-molecular-weight heparin, factor XI inhibitors, bleeding risk

## Abstract

*Background and Objectives*: Cancer-associated thrombosis (CAT) is still a leading cause of morbidity and mortality in patients with malignancy and represents a major challenge in cardio-oncology. Although low-molecular-weight heparins (LMWH) have long been the standard of care, direct oral anticoagulants (DOACs) have broadened treatment options. However, balancing thrombotic and bleeding risks, accounting for tumor- and individual-specific characteristics, requires an individualized therapeutic approach. This review summarizes current evidence supporting personalized anticoagulation strategies in CAT. *Materials and Methods*: A structured narrative review supported by a systematic literature search was conducted. Evidence regarding LMWH, DOACs, and emerging factor XI/XIa inhibitors was critically appraised, with emphasis on clinical scenarios necessitating individualized management, including gastrointestinal, genitourinary, and intracranial malignancies, hepatocellular carcinoma, thrombocytopenia, renal impairment, drug–drug interactions, and recurrent thrombosis. *Results*: Both LMWH and DOACs are effective options for CAT, with treatment selection guided by the balance between thrombotic and bleeding risks. Among DOACs, apixaban appears to have a favorable efficacy–safety profile based on current evidence, whereas rivaroxaban and edoxaban remain suitable for carefully selected patients despite higher bleeding risk in specific settings. LMWH continues to be preferred in patients with active mucosal tumors, severe thrombocytopenia, advanced renal dysfunction, significant drug–drug interactions, or when dosing flexibility is required. Emerging evidence also points to possible pleiotropic effects of DOACs on inflammation, angiogenesis, and metastatic progression, although the results have not yet translated into proven clinical benefit. Factor XI/XIa inhibitors remain investigational, with no established role in cancer-associated thrombosis. *Conclusions*: Contemporary CAT management has shifted toward personalized anticoagulant selection based on tumor characteristics, bleeding risk, organ function, anticancer therapy, and patient-related factors. Future advances, including factor XI/XIa inhibitors and a better understanding of anticoagulants’ biological effects beyond thrombosis prevention, may further optimize individualized treatment and improve clinical outcomes.

## 1. Introduction

Cancer-associated thrombosis (CAT) is a challenging condition at the intersection of oncology, hematology, and cardiovascular medicine. Venous thromboembolism (VTE), including deep vein thrombosis (DVT) and pulmonary embolism (PE), is a major cause of morbidity and mortality in cancer patients and remains the second most common cause of death after cancer progression in those receiving systemic anticancer therapy [1]. Compared with the general population, patients with malignancy have a four- to sevenfold higher risk of developing VTE, although this risk varies by tumor type, disease stage, anticancer treatment, hospitalization, surgery, immobility, and the presence of central venous catheters [2,3].

The pathogenesis of CAT reflects the relationship between tumor biology and host factors. Malignant cells promote a hypercoagulable state through tissue factor expression, activation of platelets and endothelial cells, release of procoagulant microparticles, and inflammatory cytokine signaling. Consequently, VTE incidence is particularly high in pancreatic, gastric, and brain cancers, while lung, gynecologic, genitourinary, hematologic, and renal malignancies also carry substantial thrombotic risk [4,5]. Among patients with active cancer, the incidence of a first VTE event approaches 5.8 per 100 person-years, whereas recurrent VTE occurs at about 9.6 per 100 person-years, underscoring the persistent prothrombotic state associated with malignancy [4,5].

Despite the proven benefits of anticoagulation, therapeutic decision-making continues to be difficult because cancer patients frequently exhibit concurrent risks of thrombosis and bleeding. Tumor location, mucosal involvement, thrombocytopenia, renal or hepatic dysfunction, recent surgery, invasive procedures, drug–drug interactions, and cancer-directed therapies all affect both the efficacy and safety of anticoagulant treatment. As a result, the management of CAT goes beyond selecting an anticoagulant and requires the continuous review of thrombotic and bleeding risks, treatment tolerability, and changes in the patient’s clinical status throughout the course of cancer care [1,2,5].

This review summarizes current evidence supporting an individualized approach to anticoagulation in cancer-associated thrombosis. Rather than recommending a universally preferred anticoagulant, it examines how treatment decisions should be tailored based on tumor characteristics, thrombosis phenotype, bleeding risk, organ function, concomitant anticancer therapies, treatment duration, and patient-specific factors. The review places particular emphasis on clinical scenarios that require individualized management, such as gastrointestinal and genitourinary malignancies, brain tumors, hepatocellular carcinoma, thrombocytopenia, renal impairment, drug–drug interactions, and recurrent thrombosis.

## 2. Materials and Methods

### 2.1. Search Strategy

This structured narrative review was supported by a systematic literature search. The search and study-selection process was reported in accordance with the relevant PRISMA 2020 items [6]. Two reviewers independently searched PubMed and Scopus for English-language publications published between January 2019 and June 2026. Search terms included “cancer-associated thrombosis,” “venous thromboembolism,” “direct oral anticoagulants,” and “low-molecular-weight heparin,” together with relevant synonyms and related terms, combined using the Boolean operators AND and OR.

The search was designed to identify contemporary evidence addressing anticoagulant treatment and clinically relevant management aspects of cancer-associated venous thromboembolism. In addition to completed clinical studies, ongoing and terminated clinical trials investigating emerging anticoagulant strategies were considered when relevant to the scope of the review.

### 2.2. Eligibility Criteria and Study Selection

Eligible publications included clinical trials, non-randomized observational studies, systematic reviews and meta-analyses, and evidence-based clinical practice guidelines addressing anticoagulant treatment or clinically relevant management aspects of cancer-associated venous thromboembolism in adults. Non-randomized evidence included prospective and retrospective observational studies, real-world studies, and registry-based studies. Ongoing and terminated clinical trials investigating emerging anticoagulant strategies were also considered eligible for descriptive assessment, even when final outcome data were unavailable.

Narrative reviews, expert opinion articles, editorials, case reports, conference abstracts, publications outside the predefined search period, and studies not directly relevant to CAT management were excluded from the primary qualitative synthesis. Selected preclinical and mechanistic studies were considered separately when relevant to provide biological context regarding the potential effects of DOACs and FXI/FXIa inhibition and were not included in the PRISMA study count.

**Figure 1:**
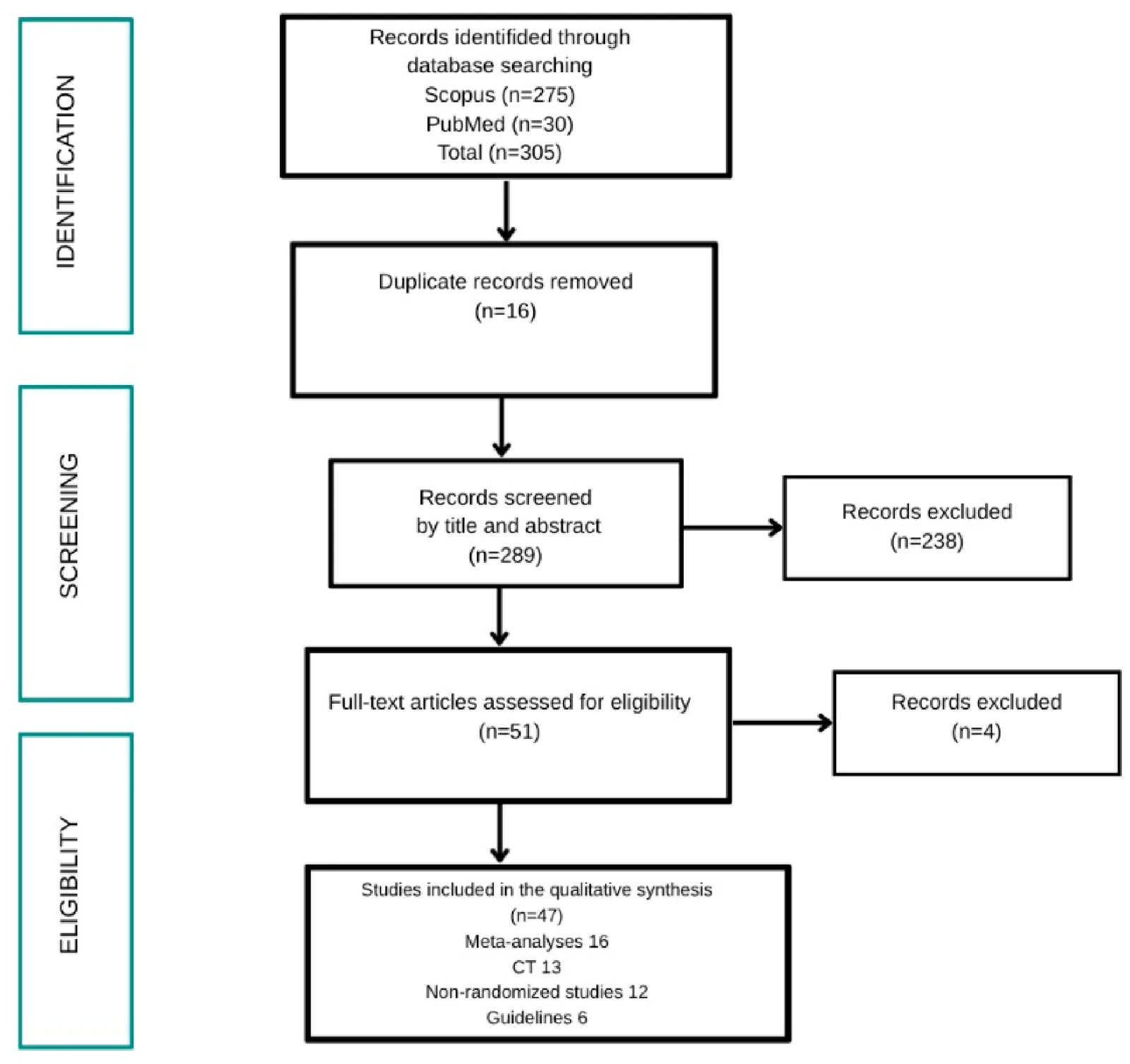
PRISMA flow diagram illustrating the study selection process.

The search identified 305 records: 275 from Scopus and 30 from PubMed. After removal of 16 duplicates, 289 records underwent title and abstract screening. Of these, 238 were excluded, and 51 full-text articles were assessed for eligibility. Four full-text articles were excluded after assessment, resulting in 47 publications being included in the qualitative synthesis: 13 clinical trials, 12 non-randomized observational studies, 16 systematic reviews and meta-analyses, and six clinical practice guidelines. Study selection was performed independently by two reviewers, with disagreements resolved by consensus. The study-selection process is summarized in Figure 1.

### 2.3. Data Extraction and Evidence Synthesis

Relevant data were extracted from the included publications according to study design and status. For completed clinical trials and non-randomized observational studies, extracted information included study design, study population, interventions and comparators, efficacy outcomes, and safety outcomes. For systematic reviews and meta-analyses, the principal pooled efficacy and safety findings were considered, whereas clinical practice guidelines were evaluated with respect to their main therapeutic recommendations and areas of consensus or divergence.

For ongoing or terminated clinical trials without available final outcome data, information on study design, study population, interventions and comparators, planned outcomes, and trial status was extracted and summarized descriptively. These studies were used to characterize emerging therapeutic strategies and the evolving evidence base but were not included in the synthesis of clinical efficacy and safety outcomes.

Given the methodological and clinical heterogeneity of the included evidence, findings were synthesized narratively rather than quantitatively pooled. Evidence was interpreted according to study design, clinical context, and availability of outcome data. Preclinical and mechanistic evidence used exclusively to provide biological context was analyzed separately and was not included in the PRISMA study count.

### 2.4. Risk-of-Bias Assessment

The methodological quality of the primary clinical studies included in the qualitative synthesis was assessed using design-specific risk-of-bias tools. Randomized controlled trials were evaluated using the revised Cochrane Risk of Bias tool (RoB 2), which assesses bias arising from the randomization process, deviations from intended interventions, missing outcome data, measurement of the outcome, and selection of the reported result. Non-randomized studies of interventions were assessed using the Risk of Bias In Non-randomized Studies of Interventions (ROBINS-I) tool. Risk-of-bias assessments were performed independently by two reviewers, with disagreements resolved by consensus.

Risk-of-bias assessments were used to inform the interpretation and weighting of the evidence rather than as an exclusion criterion. Formal outcome-specific risk-of-bias assessment was not performed for ongoing or terminated trials without available clinical outcome data.

## 3. Results

### 3.1. Characteristics of the Included Studies

The qualitative synthesis included 47 publications: 13 clinical trials, 12 non-randomized observational studies, 16 systematic reviews and meta-analyses, and 6 clinical practice guidelines. The included evidence covered randomized and non-randomized evaluations of anticoagulant therapy, emerging anticoagulant strategies, and contemporary guideline recommendations for the management of cancer-associated thrombosis.

### 3.2. Evidence from Clinical Trials

#### 3.2.1. Evidence from Randomized Clinical Trials of Anticoagulant Therapy in Cancer-Associated Thrombosis

The CLOT, Hokusai-VTE Cancer, and Anticoagulation Therapy in SELECTeD Cancer Patients at Risk of Recurrence of Venous Thromboembolism (SELECT-D) trials provided the historical foundation for the treatment of cancer-associated thrombosis. CLOT established dalteparin as more effective than vitamin K antagonist-based therapy for avoiding recurrent VTE, without a significant increase in major bleeding. Hokusai-VTE Cancer subsequently demonstrated the noninferiority of edoxaban to dalteparin, although edoxaban was associated with more major bleeding, particularly in patients with gastrointestinal malignancies. SELECT-D reported fewer recurrent VTE events with rivaroxaban than with dalteparin but a higher incidence of clinically relevant non-major bleeding. Because these landmark trials were published before 2019, they were used only to provide historical background and were not included in the PRISMA qualitative synthesis [7,8,9].

Contemporary evidence supports the use of DOACs in appropriately selected patients with CAT. In Apixaban and Dalteparin in Active Malignancy-Associated Venous Thromboembolism (ADAM VTE), apixaban was associated with low rates of recurrent VTE and major bleeding compared with dalteparin. The larger Apixaban for the Treatment of Venous Thromboembolism in Patients With Cancer (CARAVAGGIO) trial confirmed that apixaban was noninferior to dalteparin for avoiding recurrent VTE without increasing major bleeding. Cancer-Associated Thrombosis, A Pilot Treatment Study Using Rivaroxaban (CASTA-DIVA) also produced results consistent with the efficacy and safety of rivaroxaban, although the study was underpowered to demonstrate noninferiority because of its limited sample size [10,11,12].

The pragmatic Direct Oral Anticoagulants (DOACs) Versus LMWH ± Warfarin for VTE in Cancer: A Randomized Effectiveness (CANVAS) trial demonstrated that DOAC-based therapy was noninferior to LMWH-based therapy for avoiding recurrent VTE over six months, with similar rates of major bleeding [13].

Recent trials have also addressed extended anticoagulation. Extending Venous Thromboembolism Secondary Prevention with Apixaban in Cancer Patients (EVE) found similar rates of recurrent clotting events with reduced-dose and full-dose apixaban, without a statistically significant reduction in the composite bleeding endpoint. APIxaban Cancer Associated Thrombosis (API-CAT) subsequently demonstrated that reduced-dose apixaban was noninferior to full-dose apixaban for avoiding recurrent VTE and was associated with fewer clinically relevant bleeding events throughout extended treatment [14,15,16].

Rivaroxaban in the Treatment of Venous Thromboembolism (VTE) in Cancer Patients—A Randomized Phase III Study (CONKO-011) additionally showed that rivaroxaban reduced patient-reported treatment burden compared with injectable LMWH, although clinical efficacy and safety outcomes were secondary endpoints [17,18].

Overall, these outcomes support an individualized approach to anticoagulant selection. DOACs, particularly apixaban, represent effective alternatives to LMWH, whereas LMWH remains preferable in patients with active gastrointestinal or genitourinary mucosal tumors, severe thrombocytopenia, clinically important drug–drug interactions, or a high risk of bleeding. The principal contemporary randomized trials of anticoagulation in CAT are summarized in Table 1.

#### 3.2.2. Factor XI/XIa Inhibitors: A New Direction in Anticoagulation for Patients with Cancer

An important emerging direction in anticoagulant development is the inhibition of factor XI (FXI) and its activated form, FXIa. Interest in this therapeutic strategy stems from the potential to dissociate pathological thrombosis from physiological hemostasis. Unlike conventional anticoagulants, which interfere with central steps in thrombin generation and may increase bleeding risk, FXI appears to play a predominant role in the amplification and propagation of coagulation, with a less prominent role in the initiation of hemostasis [19,20].

Coagulation is initiated mainly through the tissue factor pathway, whereas FXI contributes mainly to subsequent amplification. Activated by factor XIIa (FXII) through the contact pathway or by thrombin through a positive feedback mechanism, FXIa activates factor IX (FIX) and sustains thrombin generation. Thus, FXI/FXIa inhibition may limit thrombus propagation without completely suppressing initial hemostasis. Congenital FXI deficiency, which is associated with a reduced thrombotic risk and a generally moderate bleeding phenotype, provides biological support for the development of FXI/FXIa inhibitors as anticoagulants with a potentially more favorable bleeding profile [19,20,21,22,23].

This strategy is particularly attractive in cancer, which is characterized by a persistent procoagulant state. Tumor cells, endothelial and platelet activation, inflammation, neutrophil extracellular traps (NETs), extracellular deoxyribonucleic acid (DNA), histones, and polyphosphates may promote coagulation and activation of the contact pathway. In this setting, FXI/FXIa inhibition could potentially reduce the risk of VTE and recurrence without a proportional increase in bleeding. In addition, experimental evidence suggests that FXI may be involved in inflammation, vascular permeability, tumor cell adhesion and migration, angiogenesis, and metastasis. However, any potential beneficial effect on tumor biology remains hypothetical and has not been demonstrated clinically [19,20,21,22,24].

Abelacimab was among the most advanced agents investigated for CAT. This monoclonal antibody inhibits both FXI and FXIa. Its phase III program included ASTER, which compared abelacimab with apixaban in patients with CAT considered suitable for DOAC therapy, and MAGNOLIA, which compared abelacimab with dalteparin in patients with gastrointestinal or genitourinary cancers at increased bleeding risk. The program was discontinued in 2026. ASTER was stopped following an interim assessment showing a low probability of meeting its therapeutic effect objective relative to apixaban, with no reported safety signal to drive the decision. MAGNOLIA was subsequently discontinued as part of the same strategic decision, without definitive results demonstrating inferiority to dalteparin. Thus, although abelacimab remains important in establishing the clinical concept of FXI/FXIa inhibition, it currently has no established role in the treatment of CAT [25,26].

Research is continuing with REGN7508 and REGN9933, two fully human monoclonal antibodies that target the FXI/FXIa axis through different mechanisms. REGN7508 affords broader inhibition of FXI/FXIa, whereas REGN9933 more selectively interferes with activated coagulation factor XII (FXIIa)—mediated FXI activation. REGN7508 is being investigated in the ROXI-CAT program for both the prevention and treatment of CAT. ROXI-CAT-I evaluates primary thromboprophylaxis in patients with solid tumors at increased thrombotic risk, whereas ROXI-CAT-II assesses the treatment and secondary prevention of VTE in comparison with apixaban. These studies will help determine whether FXI inhibition can maintain antithrombotic efficacy while reducing bleeding risk [27,28].

Another area of interest is catheter-related thrombosis. The ROXI-CATH study appraises REGN7508 and REGN9933 for the prevention of thrombotic complications associated with PICC insertion in patients with cancer, a setting in which activation of the contact pathway may be particularly relevant. Gruticibart, another anti-FXI monoclonal antibody that predominantly inhibits FXIIa-mediated FXI activation, has shown favorable tolerability and a possible signal for reducing catheter-related thrombosis in preliminary studies, although larger randomized trials are required [29].

Beyond their anticoagulant effects, DOACs may exert anti-inflammatory and antineoplastic effects by modulating pathways involved in tumor growth, angiogenesis, invasion, and metastasis. However, these effects have been demonstrated mainly in preclinical studies and have not been confirmed in humans; therefore, DOACs should not be considered anticancer therapies. Emerging evidence also suggests that FXI may influence inflammation, vascular permeability, tumor-cell adhesion, migration, and angiogenesis, although the clinical relevance of these effects remains uncertain.

At present, FXI/FXIa inhibitors remain investigational and are not alternatives to standard LMWH or DOAC therapy for CAT. The experience with abelacimab illustrates that the biological rationale for FXI inhibition does not necessarily translate into clinical superiority. Ongoing studies must determine whether these agents can reduce bleeding without impairing antithrombotic efficacy. If confirmed, FXI/FXIa inhibition could represent an important step toward more personalized prevention and treatment of CAT. A summary of clinical studies evaluating FXI/FXIa-targeting monoclonal antibodies in CAT is presented in Table 2.

#### 3.2.3. Relevant Non-Randomized/Real-World Evidence

Non-randomized, real-world studies complemented the randomized evidence by providing data from broader, more heterogeneous cancer populations encountered in routine clinical practice. Overall, these studies contextualized the effectiveness and safety of anticoagulant strategies under real-world conditions. Given their methodological limitations and the risk of confounding inherent to observational designs, their findings were interpreted as supportive rather than confirmatory evidence.

### 3.3. Risk-of-Bias Assessment

#### 3.3.1. Risk-of-Bias Assessment of Randomized Clinical Trials

The methodological quality of the randomized clinical trials included in the qualitative synthesis was assessed using the Cochrane Risk of Bias 2 (RoB 2) tool. Risk of bias was evaluated across the five standard domains: the randomization process, deviations from intended interventions, missing outcome data, measurement of the outcome, and selection of the reported result. Domain-level and overall judgments are presented in Table 3 and Table 4.

Overall, the completed randomized trials showed a generally favorable risk-of-bias profile, with most studies rated as low risk or presenting some concerns. The main methodological concerns involved deviations from intended interventions, missing outcome data, and selective reporting. API-CAT showed low risk across all domains, whereas CONKO-011 had a high risk of bias for the assessed patient-reported outcome. Ongoing trials without available outcome data could not be formally assessed using RoB 2.

#### 3.3.2. Risk of Bias in Non-Randomized Studies of Interventions

**Figure 2:**
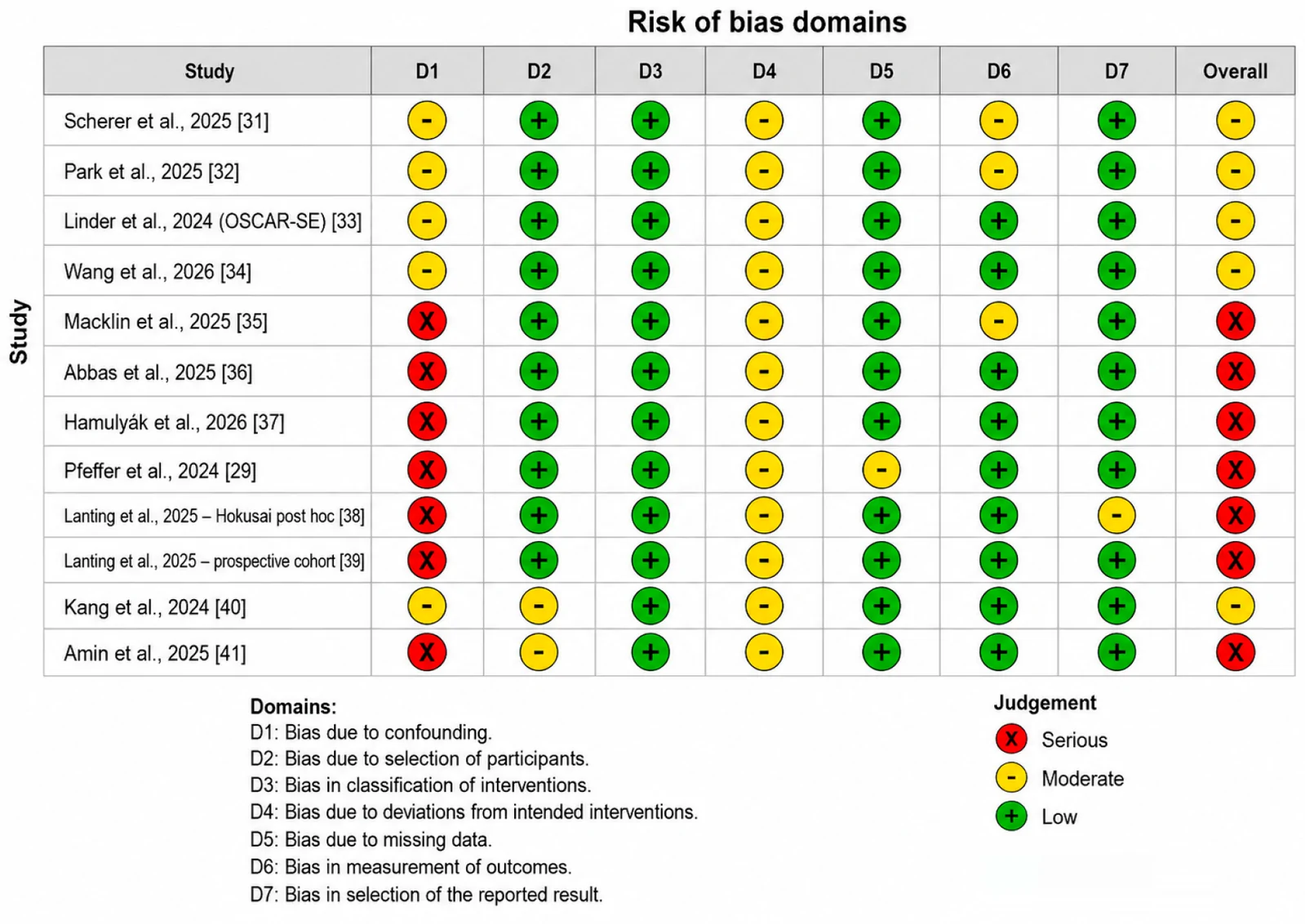
Risk-of-bias assessment of the included non-randomized studies using the ROBINS-I tool [29,31,32,33,34,35,36,37,38,39,40,41].

Risk-of-bias assessment of the non-randomized studies using ROBINS-I showed an overall moderate-to-serious risk of bias. Five studies were judged to have a moderate overall risk of bias, whereas seven were rated as having a serious risk of bias, primarily driven by concerns related to confounding. Given the narrative nature of the review, these studies were retained to provide a comprehensive representation of the available real-world evidence, while their methodological limitations were considered when interpreting and weighting their findings. Domain-level and overall judgments are presented in Figure 2.

### 3.4. Current Guideline Recommendations: Toward Personalized Anticoagulation in Cancer-Associated Thrombosis

New guidelines on CAT, including American Society of Clinical Oncology (ASCO) 2023 [41], European Society for Medical Oncology (ESMO) 2023 [42], British Society for Hematology (BSH) 2024 [43], Spanish Society of Medical Oncology (SEOM) 2024 [44], Spanish Society of Internal Medicine (SEMI) 2024/2025 [45], and National Comprehensive Cancer Network (NCCN) 2024/2025 [14,46], reflect a paradigm shift from the traditional strategy centered predominantly on LMWH toward individualized anticoagulation. Treatment selection is now based on the balance between thrombotic and bleeding risks, cancer type and activity, renal and hepatic function, drug–drug interactions, route of administration, and patient preferences [14,41,42,43,44,45,46].

The duration of anticoagulation has also become more flexible. Therapeutic anticoagulation is generally recommended for 3–6 months, after which the requirement for ongoing treatment should be reassessed periodically. Anticoagulation is usually extended while cancer remains active, anticancer treatment is ongoing, or persistent risk factors for VTE recurrence are present. Conversely, discontinuation may be considered in patients in remission who are no longer receiving active anticancer therapy and have no persistent thrombotic risk factors [42,43,44,45,46].

An important development in extended treatment is the possibility of lowering the intensity of anticoagulation after the first 6 months. The API-CAT trial demonstrated that reduced-dose apixaban (2.5 mg twice daily) was noninferior to the full dose (5 mg twice daily) for avoiding recurrent VTE and was associated with fewer clinically relevant bleeding events. This evidence supports an individualized approach to extended anticoagulation based on the patient’s evolving thrombotic and bleeding risks [14,16].

Personalization is also essential in thromboprophylaxis. Routine prophylaxis is not recommended for all ambulatory patients with cancer but may be considered in those at increased thrombotic risk and with an acceptable bleeding risk. In cancer surgery, LMWH remains the best-established standard for perioperative and extended thromboprophylaxis, whereas apixaban and rivaroxaban may represent alternatives for extended postoperative prophylaxis in selected patients [14,41,42,43,44,45,46].

Although activation of coagulation may contribute to tumor progression and metastasis, clinical studies have not demonstrated a consistent survival benefit from anticoagulant therapy in the absence of an established thrombotic or prophylactic indication. Therefore, anticoagulation is not currently recommended as an antineoplastic strategy [41,42,43,44].

Overall, current guidelines reflect a shift from a uniform LMWH-based approach to a personalized anticoagulation model. The selection of anticoagulant, treatment duration, and intensity beyond the initial six months should be dynamically tailored to the patient’s clinical profile and the progression of the underlying malignancy, aiming to optimize the balance between preventing thrombotic recurrence and minimizing bleeding risk. A summary of current guidelines and a comparative review is presented in Table 5.

## 4. Discussion

### 4.1. Personalized Anticoagulation

#### 4.1.1. Anticoagulation in Gastrointestinal Malignancies

The treatment of established VTE in patients with gastric cancer is particularly challenging because of the competing risk of anticoagulant-related bleeding. Factors such as active luminal tumors, mucosal ulceration, gastrointestinal bleeding, interventions, and treatment-related thrombocytopenia increase hemorrhagic risk. Therefore, individualize therapeutic anticoagulation based on each patient’s thrombotic and bleeding risks.

NCCN 2025 [14,46] guidelines recommend DOACs only in patients without active gastroesophageal or gastric lesions, whereas LMWH remains the preferred option for patients with upper gastrointestinal tumors, unresected lesions, or a high risk of gastrointestinal bleeding. These recommendations are supported by Nishimoto et al. (2024), who reported a significantly higher risk of bleeding with DOACs in patients with upper gastrointestinal malignancies, whereas this excess risk was not observed in colorectal cancer, illustrating the importance of tumor location when selecting anticoagulant therapy [14,47].

Recent meta-analyses, including Ren et al. (2025), indicate that DOACs lower recurrent VTE rates compared with LMWH without increasing major bleeding risk, although non-major bleeding is more frequent [48]. These results should be interpreted with caution, as patients at high risk of bleeding are often excluded from clinical trials or preferentially treated with LMWH. Real-world studies also suggest that DOACs are effective and safe in appropriately selected patients, although selection bias remains an important limitation [34,40,48,49].

Among DOACs, apixaban appears to have a favorable efficacy–safety profile based on current evidence and may be considered in patients without active luminal lesions, recent gastrointestinal bleeding, or impaired drug absorption. In contrast, rivaroxaban and edoxaban should be used with greater caution in upper gastrointestinal malignancies. For patients requiring extended anticoagulant treatment, the API-CAT trial demonstrated that reduced-dose apixaban (2.5 mg twice daily) was non-inferior to the standard dose for the prevention of recurrent VTE after the initial 6 months of anticoagulation, whereas the EVE trial did not demonstrate a clear reduction in bleeding with dose reduction [14,15,16].

Factor XI/XIa inhibitors remain under investigation. Although the phase III MAGNOLIA trial evaluated abelacimab versus dalteparin in cancer-associated thrombosis, the unfavorable findings from the ASTER trial led to discontinuation of its clinical development. Therefore, FXI/XIa inhibitors cannot currently be recommended as validated alternatives to LMWH or DOACs for the treatment of VTE in patients with gastric cancer [19,26].

Overall, anticoagulant treatment of established VTE in patients with gastrointestinal malignancies should be individualized based on tumor location, disease status, and thrombotic and bleeding risk. LMWH remains the preferred option in patients with active or unresected upper gastrointestinal tumors, particularly gastric cancer, and in those at high risk of gastrointestinal bleeding. DOACs, particularly apixaban, may be considered in carefully selected patients with a low-to-moderate bleeding risk, including patients with gastric cancer without active luminal lesions or recent gastrointestinal bleeding. Their use may be particularly appropriate in colorectal cancer, where the excess bleeding risk associated with DOACs appears to be less pronounced than in upper gastrointestinal malignancies. Given their oral administration and greater ease of use compared with LMWH, DOACs may also offer practical advantages when extended anticoagulant treatment is required. In appropriately selected patients who have completed at least six months of therapeutic anticoagulation and remain candidates for extended treatment, reduced-dose apixaban may be considered for extended secondary prevention of recurrent VTE, provided gastrointestinal bleeding risk is not high and thrombotic and bleeding risks are reassessed regularly.

#### 4.1.2. Anticoagulation in Genitourinary Malignancies

The treatment of established VTE in patients with genitourinary malignancies represents a particular therapeutic challenge because the need for effective anticoagulation frequently coexists with an increased risk of site-specific bleeding. Active urothelial tumors, hematuria, transurethral procedures, nephrostomy tubes, ureteral stents, and major urological surgery all influence anticoagulant selection and require an individualized assessment of thrombotic and bleeding risks.

LMWH remains the preferred anticoagulant in patients with established VTE and active hematuria, unresected urothelial tumors, recent transurethral resection of bladder tumors (TURBT), nephrostomy tubes, ureteral stents, or other situations associated with a high risk of bleeding. In patients with stable hemostasis and a lower risk of genitourinary bleeding, DOACs may be considered on an individualized basis. Yang et al. (2025) also noted that anticoagulant and antiplatelet therapies may precipitate gross hematuria, which can lead to the diagnosis of previously unrecognized urological malignancies [31,50,51].

Renal cell carcinoma with tumor thrombus represents a distinct clinical entity. Although these patients are at increased risk of VTE, current evidence indicates that anticoagulation alone does not result in tumor thrombus regression, which primarily depends on surgical and oncologic treatment. Therefore, anticoagulation should not be routinely prescribed for tumor thrombus itself but should be reserved for patients with concomitant bland venous thrombosis, established VTE, or another independent indication for anticoagulant therapy [52,53].

Factor XI/FXIa inhibitors remain investigational in cancer-associated thrombosis. Although the MAGNOLIA trial evaluated abelacimab versus dalteparin, the development program was discontinued following an unfavorable interim efficacy assessment in ASTER, without a reported safety signal driving the decision. Therefore, these agents cannot currently be recommended for routine VTE treatment in patients with genitourinary malignancies [19,26].

Overall, LMWH remains the preferred anticoagulant for patients with genitourinary malignancies and established VTE when the risk of genitourinary bleeding is high, particularly with active hematuria or locally active urothelial disease. Consider DOACs in carefully selected patients with stable hemostasis and no active bleeding. In renal cell carcinoma with tumor thrombus, anticoagulation should be individualized and reserved for patients with concomitant venous thromboembolism or another independent indication for anticoagulant therapy.

#### 4.1.3. Anticoagulation in Brain Tumors

The management of VTE in patients with primary or metastatic brain tumors remains one of the most challenging clinical scenarios in cancer-associated thrombosis. These patients are simultaneously at high risk of recurrent thromboembolic events and intracranial hemorrhage (ICH), making anticoagulant therapy a fine balance between thrombotic protection and neurological safety.

Brain tumors are no longer considered an absolute contraindication to therapeutic anticoagulation. Treatment should be individualized based on bleeding and thrombotic risk, recent neurosurgery, platelet count, thrombotic burden, and neurological status. As emphasized by Ranjan et al. contemporary management relies on a risk-stratified rather than a restrictive approach to anticoagulation [54,55].

Recent studies suggest that DOACs have an intracranial bleeding risk comparable to that of LMWH. In patients with glioma and glioblastoma, Amin et al. (2025) [32] found no significant difference in the incidence of intracranial hemorrhage between DOACs and LMWH, although fatal bleeding events occurred only in the LMWH group. Similarly, the ABC study by Hamulyak et al. (2026) reported a lower cumulative 12-month incidence of spontaneous intracranial hemorrhage with DOACs than with LMWH (6.8% vs. 13%), although this difference did not reach statistical significance [32,37].

The management of brain metastases requires additional consideration because the risk of spontaneous intracranial hemorrhage varies according to the primary tumor. As summarized by Hamulyak et al. (2024), melanoma, renal cell carcinoma, choriocarcinoma, thyroid carcinoma, and hepatocellular carcinoma are associated with the highest hemorrhagic risk [56]. In this setting, DOACs and LMWH appear to have comparable safety profiles, and anticoagulant selection should be guided by the biology of the primary malignancy, radiological characteristics of the brain lesions, and the overall bleeding risk [37,56].

Therapeutic anticoagulation is appropriate for acute VTE when thrombotic risk exceeds intracranial bleeding risk. DOACs, particularly apixaban, may be considered in stable glioma or glioblastoma without active bleeding, recent high-risk neurosurgery, or severe thrombocytopenia. LMWH remains preferable when bleeding risk is high or rapid dose adjustment is required. In brain metastases, treatment should be individualized, especially for tumors with a high spontaneous bleeding risk [32,37,54,55,56].

Factor XI/XIa inhibitors remain investigational in this setting. Although abelacimab has been evaluated in phase III programs for cancer-associated thrombosis, patients with primary brain tumors and intracranial metastases were excluded from the ASTER trial, while the MAGNOLIA trial focused on patients with gastrointestinal and genitourinary malignancies. Consequently, there is currently no evidence supporting the use of factor XI/XIa inhibitors in patients with brain tumors and cancer-associated thrombosis [19,26].

#### 4.1.4. Anticoagulation in Hepatocellular Carcinoma

Patients with hepatocellular carcinoma (HCC) are at increased risk of VTE, particularly portal vein thrombosis, while anticoagulation continues to be challenging because of the concomitant high risk of bleeding. Current evidence supports the use of LMWHs or DOACs, particularly apixaban and edoxaban, in patients with preserved liver function (Child–Pugh A and selected Child–Pugh B), as these agents have exhibited efficacy equivalent to or better than vitamin K antagonists in achieving portal vein recanalization, with a similar or lower risk of major bleeding [57,58].

For HCC-associated thrombosis, the choice of DOAC should reflect the degree of underlying hepatic impairment. In patients with Child–Pugh A cirrhosis, apixaban and edoxaban may be considered when there is a clear indication for anticoagulation and no major bleeding contraindications. In patients with Child–Pugh B cirrhosis, including those with HCC, apixaban and edoxaban may be considered with particular caution following individualized assessment of thrombotic and bleeding risks [59,60].

For apixaban, no dose adjustment is required solely on the basis of mild or moderate hepatic impairment according to European prescribing information; however, clinical experience in patients with Child–Pugh B cirrhosis remains limited. Avoid or defer anticoagulation in the presence of active or recent major bleeding, untreated gastroesophageal varices at high risk of bleeding, or other major bleeding risk factors, while severe thrombocytopenia requires individualized decision-making. Consider evaluation and appropriate management of gastroesophageal varices before initiating anticoagulation when clinically indicated [59].

In patients with Child–Pugh C cirrhosis, DOACs are not routinely recommended, and the choice of anticoagulant should be individualized [59].

HCC itself does not constitute an absolute contraindication to DOAC therapy; however, it warrants additional assessment of bleeding risk, as observational data suggest an increased risk of major bleeding in patients with cirrhosis and HCC receiving DOACs [61].

In patients with mixed portal vein thrombosis, anticoagulation targets only the benign thrombotic component and does not affect the tumor thrombus. However, the presence of bulky HCC, particularly tumors > 7–10 cm, with subcapsular or exophytic growth, central necrosis, vascular invasion, or marked arterial hypervascularity, substantially increases the risk of intratumoral hemorrhage and spontaneous rupture. In this setting, anticoagulation may facilitate the progression of a localized intratumoral bleed to massive hemoperitoneum and hemorrhagic shock. Therefore, the decision to initiate anticoagulation should rely on an individualized assessment that integrates tumor characteristics, the extent of portal vein thrombosis, liver function, bleeding risk, and the feasibility of locoregional treatment, rather than on the presence of mixed portal vein thrombosis alone. Although both LMWHs and DOACs are reasonable therapeutic options in appropriately selected patients, LMWHs may be considered in individuals at particularly high bleeding risk because of their shorter half-life and the possibility of rapid treatment discontinuation in the event of bleeding or before invasive procedures; however, comparative evidence supporting this approach remains limited [62,63,64,65].

#### 4.1.5. Anticoagulation in Breast Cancer

Breast cancer is generally associated with a lower thromboembolic risk than pancreatic, gastrointestinal, or lung malignancies, although VTE remains clinically relevant, particularly in patients with advanced disease and during active anticancer treatment [66,67].

In patients with established VTE and a low bleeding risk, DOACs, particularly apixaban or rivaroxaban, represent appropriate treatment options, whereas LMWH, such as enoxaparin or dalteparin, may be preferred when invasive procedures are anticipated, thrombocytopenia is present, bleeding risk is increased, or clinically relevant drug–drug interactions limit DOAC use. Unlike gastrointestinal malignancies, breast tumors are not typically associated with mucosal bleeding, making DOAC therapy more broadly applicable in appropriately selected patients [66].

Thrombotic risk in breast cancer is also influenced by anticancer treatment. Chemotherapy, tamoxifen, surgery, and CDK4/6 inhibitors may increase VTE risk. However, routine anticoagulant prophylaxis solely because of CDK4/6 inhibitor therapy is not currently supported, and prophylaxis should be individualized based on overall thrombotic and bleeding risk [67,68].

Endocrine therapy does not necessarily need to be discontinued following VTE when adequate therapeutic anticoagulation can be maintained [69].

Upper-extremity DVT (UEDVT) deserves particular consideration in breast cancer, as it may occasionally represent the first clinical manifestation of an occult malignancy. In addition to the systemic prothrombotic state associated with cancer, UEDVT may result from local tumor-related mechanisms, including extrinsic venous compression or direct vascular invasion, while central venous catheters used for anticancer therapy represent another important contributing factor. A recently reported case of occult breast cancer presenting with extensive UEDVT illustrates this mechanism, in which thrombosis was predominantly related to local tumor infiltration and vascular compression rather than systemic hypercoagulability. Management generally follows the same anticoagulation principles as for lower-extremity DVT, and an initial course of LMWH followed by transition to a DOAC may be a reasonable strategy in appropriately selected patients. In the reported case, enoxaparin followed by rivaroxaban was associated with symptom resolution and substantial thrombus regression during follow-up.

Anticoagulation should generally be continued for at least 3–6 months, with treatment beyond six months considered when cancer remains active or anticancer therapy persists. Individualize the duration and intensity of extended anticoagulation based on ongoing risk of VTE recurrence and bleeding, and reassess periodically [66].

In selected patients who have completed at least six months of therapeutic anticoagulation and remain candidates for extended treatment, reduced-dose apixaban may be considered for secondary prevention, particularly when reducing bleeding risk is clinically relevant [66,70].

#### 4.1.6. Anticoagulation in Cancer-Associated Thrombosis and Thrombocytopenia

Thrombocytopenia is a frequent complication of cancer and anticancer therapy, particularly in patients with hematologic malignancies, myelosuppressive chemotherapy, or hematopoietic stem cell transplantation. Although thrombocytopenia increases bleeding risk, it does not protect against VTE, making anticoagulant management particularly challenging.

Current evidence supports a platelet-guided, individualized approach. In patients without thrombocytopenia, both LMWH and DOACs are appropriate treatment options. However, once thrombocytopenia develops, anticoagulant management should be guided primarily by platelet count, thrombotic burden, bleeding risk, the anticipated duration of thrombocytopenia, and the underlying malignancy [71,72].

According to recent evidence, including the review by Szmit et al. (2025), three therapeutic methods may be considered: full-dose anticoagulation, dose-modified anticoagulation, or temporary interruption of treatment [72]. Therapeutic anticoagulation can generally be maintained when platelet counts exceed 50 × 10^9^/L in the absence of additional major bleeding risk factors. When platelet counts fall below this threshold, treatment should be individualized based on thrombotic risk. In patients with acute high-risk VTE, such as proximal pulmonary embolism or extensive proximal deep vein thrombosis, full-dose LMWH with platelet transfusion support to maintain platelet counts of approximately 40–50 × 10^9^/L may be appropriate. In contrast, patients with lower-risk or subacute VTE can often be managed with dose-reduced LMWH or temporary interruption of anticoagulation [71,72].

Evidence supporting DOAC use in thrombocytopenic patients remains limited. Observational studies and post hoc analyses suggest similar thrombotic outcomes with DOACs and LMWH in carefully selected patients with mild thrombocytopenia, although gastrointestinal bleeding appears more frequent with edoxaban, particularly in patients with gastrointestinal malignancies. Overall, current evidence is still insufficient to replace LMWH as the preferred anticoagulant in patients with significant thrombocytopenia [73,74].

LMWH remains the anticoagulant of choice in severe thrombocytopenia because it allows dose adjustment based on platelet count, facilitates management during invasive procedures, and provides greater flexibility in the setting of bleeding or platelet transfusion. Unfractionated heparin (UFH) may be preferred in selected unstable patients because of its short half-life, rapid reversibility, and ease of monitoring. DOACs should generally be reserved for carefully selected patients with mild thrombocytopenia and a low bleeding risk. Fondaparinux has only a limited role in this setting and should not be considered a routine alternative to LMWH. It may be considered in selected patients with contraindications to heparins, particularly those with suspected or confirmed heparin-induced thrombocytopenia, although evidence supporting its use in thrombocytopenic patients with CAT remains scarce. The ongoing Strategies for Anticoagulation in Patients with Thrombocytopenia and Cancer-Associated Thrombosis (START) pilot trial is expected to provide prospective evidence regarding platelet-guided anticoagulation strategies in patients with acute CAT and platelet counts below 50 × 10^9^/L [30].

Overall, platelet count remains the principal determinant of anticoagulant management in thrombocytopenic patients with CAT. Full-dose anticoagulation can generally be maintained when platelet counts exceed 50 × 10^9^/L. Between 25 and 50 × 10^9^/L, LMWH is generally preferred because treatment intensity can be individualized based on thrombotic and bleeding risks, with platelet transfusion considered for patients with acute high-risk VTE. UFH may be useful in selected unstable patients calling for rapid dose adjustment or reversal, whereas anticoagulation is generally withheld when platelet counts fall below 25 × 10^9^/L unless thrombosis is life-threatening. DOACs should be reserved for carefully selected patients with mild-to-moderate thrombocytopenia and a low bleeding risk, while fondaparinux should be considered only in exceptional circumstances, such as contraindications to heparins or a raised suspicion of heparin-induced thrombocytopenia [71,72,73]. Suggested anticoagulation strategies for CAT according to platelet count in patients with thrombocytopenia are shown in Table 6.

#### 4.1.7. Anticoagulation in Cancer-Associated Thrombosis and Renal Impairment

Renal impairment represents a major challenge in the management of CAT because it increases both thrombotic and bleeding risks. Reduced kidney function alters the pharmacokinetics of anticoagulants, particularly LMWH and DOACs, increasing the risk of drug accumulation and hemorrhagic complications. Anticoagulant therapy should therefore be individualized according to creatinine clearance (CrCl), preferably estimated using the Cockcroft–Gault equation, while additionally considering thrombotic burden, bleeding risk, cancer type, concomitant anticancer therapy, and the dynamic course of renal function.

Evidence supporting anticoagulant therapy in patients with severe renal impairment remains limited because these patients have been largely excluded from pivotal CAT trials. For example, Laporte et al. (2024) [76] excluded patients receiving dialysis or with CrCl < 15 mL/min, and fewer than 2% of enrolled patients had CrCl < 30 mL/min. In contrast, patients with moderate renal impairment were included in the Caravaggio trial, where apixaban was not associated with an increased risk of major bleeding compared with dalteparin [33,76,77].

Management becomes more complex when CrCl falls below 30 mL/min. Current guidelines generally favor UFH or dose-adjusted LMWH because these agents allow greater flexibility, laboratory monitoring, and dose modification. The UK Kidney Association recommends reduced initial LMWH doses with anti-factor Xa monitoring to minimize drug accumulation, while acknowledging the limited evidence available for patients with severe renal impairment [14,43,76,78].

In patients with CrCl < 15 mL/min or receiving dialysis, robust evidence remains lacking. Although apixaban has been increasingly prescribed in dialysis patients, particularly for atrial fibrillation, randomized data supporting its routine use in CAT are unavailable because these patients were largely excluded from clinical trials. Consequently, UFH is generally preferred during the acute phase because of its short half-life, reversibility, and ease of titration. LMWH may be considered in selected patients with careful dose adjustment and anti-factor Xa monitoring, whereas any use of DOACs should be individualized within a multidisciplinary team [14,43,76,79].

Additional evidence is provided by the meta-analysis of Ma et al. (2024), which, although not specific to patients with cancer, suggested that DOACs may be associated with a higher bleeding risk than LMWH during the acute treatment of VTE in patients with renal impairment, without exhibiting a clear difference in efficacy [80].

Overall, renal function should be regarded as one of the principal determinants of anticoagulant selection in CAT. Patients with CrCl ≥ 50 mL/min can generally receive standard-dose DOACs or LMWH. In those with CrCl 30–50 mL/min, DOAC therapy remains appropriate, with apixaban supported by the strongest clinical evidence, edoxaban requiring dose reduction, and rivaroxaban reserved for carefully selected patients. When CrCl declines below 30 mL/min, UFH or dose-adjusted LMWH remains the preferred therapeutic option because of their greater flexibility and the limited evidence supporting DOAC use. Although observational studies suggest that reduced-doses apixaban may be a reasonable option in carefully selected patients with advanced chronic kidney disease or dialysis, current evidence is insufficient to recommend its routine use in CAT. Therefore, anticoagulant therapy should be individualized and renal function reassessed regularly throughout cancer treatment.

#### 4.1.8. Drug–Drug Interactions Between Anticoagulants and Anticancer Therapies

Drug–drug interactions are an important determinant of anticoagulant selection in patients with CAT. DOACs are substrates of P-glycoprotein (P-gp), whereas apixaban and rivaroxaban are also partially metabolized by cytochrome P450 3A4 (CYP3A4). Consequently, strong inhibitors of P-gp or CYP3A4 may increase DOAC exposure and bleeding risk, whereas enzyme inducers may reduce anticoagulant concentrations and increase the risk of recurrent thrombosis. In contrast, LMWH have minimal pharmacokinetic interactions and therefore remain the preferred anticoagulants in patients receiving multiple anticancer agents, with unstable renal or hepatic function, or receiving therapies with high potential for interaction [81,82].

Conventional chemotherapy agents generally do not directly interact with DOACs. However, chemotherapy-related diarrhea, vomiting, thrombocytopenia, hepatic dysfunction, or nephrotoxicity may affect DOAC absorption or clearance and increase bleeding or thrombotic risk; temporary LMWH may therefore be preferable. Fluoropyrimidines, particularly capecitabine and 5-fluorouracil, can markedly increase the anticoagulant effect of warfarin, so this combination should be avoided or managed with close INR monitoring [81,83,84,85].

Targeted therapies frequently produce clinically relevant interactions with anticoagulants. Among androgen receptor pathway inhibitors used in prostate cancer, enzalutamide and apalutamide have the highest potential for interaction due to CYP3A4 and P-gp induction, which can reduce DOAC exposure. Although recent clinical data suggest that these pharmacokinetic interactions do not always translate into worse clinical outcomes, careful monitoring or temporary LMWH therapy should be considered in frail patients, those with renal impairment, or individuals receiving multiple interacting medications [35,86].

Antiangiogenic therapies, including bevacizumab and vascular endothelial growth factor receptor tyrosine kinase inhibitors (VEGFR-TKIs) such as sunitinib, pazopanib, cabozantinib, axitinib, lenvatinib, and regorafenib, primarily interact through pharmacodynamic mechanisms rather than pharmacokinetic pathways. By impairing endothelial integrity, angiogenesis, and vascular repair, these agents increase bleeding risk independently of anticoagulant plasma concentrations. Therapeutic anticoagulation is not contraindicated but should be administered cautiously in patients with active bleeding lesions, uncontrolled hypertension, recent surgery, or other conditions associated with a high hemorrhagic risk [32,55,87].

Some targeted therapies also increase bleeding risk through hematologic toxicity. Poly(ADP-ribose) polymerase (PARP) inhibitors, including olaparib, niraparib, and rucaparib, may induce anemia and thrombocytopenia, thereby increasing the bleeding risk during anticoagulant therapy. Likewise, temozolomide and lomustine, commonly used in high-grade gliomas, have minimal pharmacokinetic interactions with DOACs but frequently cause myelosuppression and thrombocytopenia, making anticoagulant management primarily dependent on platelet count rather than drug interactions [88,89,90].

Patients with primary or metastatic brain tumors represent a notably difficult population because of their intrinsic risk of intracranial hemorrhage. Bevacizumab, frequently used in recurrent glioblastoma, further increases vascular fragility through anti-vascular endothelial growth factor (VEGF) activity, while enzyme-inducing antiepileptic drugs such as carbamazepine, phenytoin, and phenobarbital may substantially reduce DOAC concentrations through CYP3A4 and P-gp induction. In contrast, levetiracetam and lacosamide have a considerably lower potential for interaction and are generally preferred when concomitant anticoagulation is required. In patients receiving strong enzyme-inducing antiepileptic drugs, LMWH or UFH is usually preferred because of their predictable pharmacokinetics [32,55,91,92].

Strong CYP3A4 and P-gp inhibitors, including azole antifungals such as fluconazole, may increase DOAC exposure and have been associated with clinically relevant bleeding, underscoring the need to evaluate all concomitant medications before initiating anticoagulant therapy [81].

Overall, systematic assessment of drug–drug interactions should be an integral part of anticoagulant selection in CAT. Pharmacokinetic interactions are most relevant for DOACs and vitamin K antagonists (VKAs), whereas pharmacodynamic interactions primarily increase bleeding risk without necessarily altering anticoagulant concentrations. LMWH remains the preferred anticoagulant in patients receiving multiple interacting drugs, strong CYP3A4/P-gp modulators, antiangiogenic therapies, significant gastrointestinal toxicity, thrombocytopenia, or enzyme-inducing antiepileptic drugs. DOACs remain appropriate for carefully selected patients without clinically relevant interactions and with stable renal and hepatic function.

#### 4.1.9. Catheter-Related Thrombosis

Catheter-related thrombosis (CRT) is a common manifestation of CAT, indicating the widespread use of central venous catheters (CVCs) for chemotherapy, parenteral nutrition, transfusions, and supportive care. CRT results from the combined effects of endothelial injury, venous stasis, catheter-related factors, and the cancer-associated hypercoagulable state [93,94,95].

The incidence of CRT varies widely according to catheter type, patient population, and diagnostic strategy, with asymptomatic events likely underestimated because routine ultrasound screening is uncommon. Current evidence reliably demonstrates a higher thrombotic risk with peripherally inserted central catheters (PICCs) than with totally implantable venous ports. Consequently, implanted ports should be preferred whenever feasible in patients requiring long-term systemic therapy, particularly those at high thrombotic risk [93,94,96].

Prospective data from the TESEO registry confirmed that CRT accounts for approximately 10% of CAT events and is predominantly symptomatic, whereas pulmonary embolism and major bleeding are relatively uncommon. CRT is a multifactorial complication influenced by cancer-related factors, including metastatic disease, previous VTE, thrombophilia, systemic inflammation, infection, thrombogenic anticancer therapy, and by device-related characteristics such as PICC use, large catheter-to-vein ratio, multiple insertion attempts, catheter dysfunction, catheter-related infection, and catheter-tip malposition [97].

CRT should be suspected in patients presenting with unilateral upper-extremity swelling, pain, venous congestion, erythema, collateral veins, or catheter dysfunction. Compression ultrasonography remains the first-line diagnostic test, whereas computed tomography or magnetic resonance venography should be considered when central venous thrombosis is suspected despite inconclusive ultrasound findings [94,95].

Therapeutic anticoagulation continues to be the cornerstone of treatment. Current evidence suggests comparable efficacy and safety of DOACs and LMWH in selected patients, although LMWH is generally preferred in patients with symptomatic CRT, gastrointestinal or genitourinary malignancies, thrombocytopenia, renal impairment, active bleeding, or significant drug–drug interactions. Anticoagulation should be continued for at least 3 months and for as long as the catheter remains in place if it is functional, uninfected, correctly positioned, and still required. Catheter removal should be reserved for catheter-related infection, persistent dysfunction, thrombus progression despite anticoagulation, or persistent symptoms [94,95,98,99].

Routine pharmacological thromboprophylaxis is not recommended. Prevention should instead focus on appropriate catheter selection, preferential use of implanted ports when feasible, optimal insertion technique, correct catheter-tip positioning, infection prevention, and prompt removal of unnecessary catheters [93,94,95,96].

Overall, CRT requires an individualized management strategy that balances the preservation of central venous access with thrombotic and bleeding risks. Management should consider catheter characteristics, cancer status, platelet count, renal and hepatic function, and the patient’s overall clinical condition.

### 4.2. Recurrent Cancer-Associated Thrombosis During Anticoagulant Therapy

#### 4.2.1. Management of Recurrent Cancer-Associated Thrombosis

Recurrent VTE in spite of ongoing anticoagulant therapy constitutes one of the most challenging clinical scenarios in the management of CAT. Recurrence may reflect progression of the underlying malignancy or failure of anticoagulant therapy due to inadequate dosing, poor adherence, impaired gastrointestinal absorption, drug–drug interactions, or mechanical factors such as tumor-related vascular compression. Therefore, before modifying anticoagulant treatment, recurrent VTE should be objectively confirmed, and potentially reversible causes of anticoagulation failure should be investigated.

Current guidelines recommend a systematic evaluation of anticoagulant adherence, concomitant medications, renal function, and, when appropriate, anticoagulant plasma levels, particularly in patients receiving DOACs. Other causes of recurrent thrombosis, including heparin-induced thrombocytopenia or antiphospholipid syndrome, should also be considered. The 2025 NCCN Guidelines define recurrent CAT as either progression of an existing thrombus or the development of a new episode of DVT or PE during therapeutic anticoagulation, and recommend reassessing patient adherence, potential drug interactions, and anticoagulant exposure before treatment escalation [14,100,101].

Despite increasing clinical experience, the optimal management of recurrent CAT remains insufficiently standardized. The prospective study by Lanting et al. (2025) demonstrated that patients with recurrent VTE remain at substantial risk of both further thrombotic events and clinically relevant bleeding despite modification of anticoagulant therapy [39]. Similar findings were reported in the post hoc analysis of the Hokusai VTE Cancer trial, where recurrent VTE, major bleeding, and mortality remained frequent despite anticoagulant dose escalation or switching between treatment strategies. These findings highlight that recurrent CAT often reflects persistent cancer-associated hypercoagulability rather than inadequate anticoagulation alone [14,100,101].

Current guidelines converge on multiple practical management principles. In patients who develop recurrent VTE while receiving a DOAC, switching to therapeutic-dose LMWH is generally recommended because evidence supporting DOAC dose escalation is lacking. In patients already receiving LMWH, dose escalation or switching to an alternative parenteral anticoagulant may be considered. For patients treated with UFH, anticoagulant intensity should be reassessed using activated partial thromboplastin time (aPTT) or anti-factor Xa activity, followed by dose adjustment or a change in anticoagulant therapy if necessary. Over the course of this process, treatment decisions should take into account bleeding risk, renal function, platelet count, tumor location, and planned invasive procedures [14,38,39].

Overall, recurrent CAT during anticoagulant therapy should not be regarded simply as a treatment failure but as a complex medical event that demands a comprehensive review of both the patient and the anticoagulant strategy. Current evidence supports switching from DOACs to LMWH after recurrence, whereas escalation of LMWH remains the preferred approach for recurrence during LMWH therapy. Because both recurrent thrombosis and bleeding remain common despite treatment modification, management should be individualized within a multidisciplinary team.

#### 4.2.2. Predictors and Risk Assessment Models for Recurrent Cancer-Associated Thrombosis

Accurate prediction of recurrent VTE in patients with CAT continues to be challenging because recurrence is affected by several factors, including cancer activity, tumor type, metastatic burden, anticancer treatment, patient characteristics, and the clinical features of the index thrombotic event. Consequently, no risk assessment model currently provides sufficient accuracy to independently direct therapeutic decisions [101,102,103].

A recent meta-analysis by Khan et al. identified several independent predictors of recurrent VTE during anticoagulant therapy. Previous VTE, impaired performance status, advanced or metastatic disease, and pancreatic, hepatobiliary, lung, and genitourinary cancers were consistently associated with an increased risk of recurrence. In contrast, breast cancer, recent surgery, female sex, and older age were associated with a lower recurrence risk. However, these predictors should always be interpreted together with bleeding risk, particularly in patients with gastrointestinal, genitourinary, or intracranial malignancies [102].

The Ottawa score is the best-known model expressly developed to predict recurrent CAT during the first six months of anticoagulation. It incorporates female sex, lung cancer, and previous VTE as risk factors, whereas breast cancer and stage I disease are considered protective factors. Although both the original version and modified Ottawa scores have been externally validated, their overall discriminatory performance remains modest, indicating that they should not be used alone to guide anticoagulant dose escalation, de-escalation, or discontinuation [101,104].

The Caravaggio score, derived from the Caravaggio trial and externally validated in the SEOM (TESEO) Thrombosis and Neoplasia registry, represents the most recent clinical prediction model for recurrent CAT. It incorporates six readily available variables: symptomatic VTE, ovarian or uterine cancer, pancreatic cancer, metastatic disease, adenocarcinoma histology, and active treatment with platinum- or fluoropyrimidine-based chemotherapy. The score stratifies patients into low-, intermediate-, and high-risk categories and appears particularly useful for identifying patients at low risk of recurrence, rather than for guiding anticoagulant selection or dose adjustment [105].

Artificial intelligence (AI) and machine-learning models represent an emerging field in recurrence prediction. Models such as Predict AI integrate clinical, laboratory, and oncological variables and have demonstrated better discrimination than conventional clinical scores in preliminary studies. Nevertheless, these tools require prospective validation before they can be incorporated into routine clinical practice [106].

From a clinical perspective, recurrence-risk models should be regarded as complementary decision-support tools rather than determinants of anticoagulant management. They may help identify patients who require closer follow-up and prolonged anticoagulation, particularly beyond the initial 6 months of treatment. However, decisions regarding anticoagulant selection, dose modification, treatment escalation, or discontinuation should not rely on recurrence-risk scores alone but should integrate tumor characteristics, cancer activity, bleeding risk, renal and hepatic function, platelet count, patient preferences, and the expected course of the underlying malignancy [101,102].

Unlike recurrence prediction, no bleeding-risk score has been universally adopted for patients with CAT. Although models such as bleeding risk prediction score for patients with venous thromboembolism (VTE-BLEED) and the Computerized Registry of Venous Thromboembolic Disease (RIETE) Bleeding Score have been evaluated, current international guidelines continue to recommend an individualized assessment of bleeding risk based on tumor site, thrombocytopenia, renal and hepatic function, previous bleeding, concomitant medications, and planned invasive procedures rather than the routine use of a formal bleeding-risk score [107,108]. Clinical risk evaluation models used in CAT are shown in Table 7.

### 4.3. Practical Clinical Algorithm

**Figure 3:**
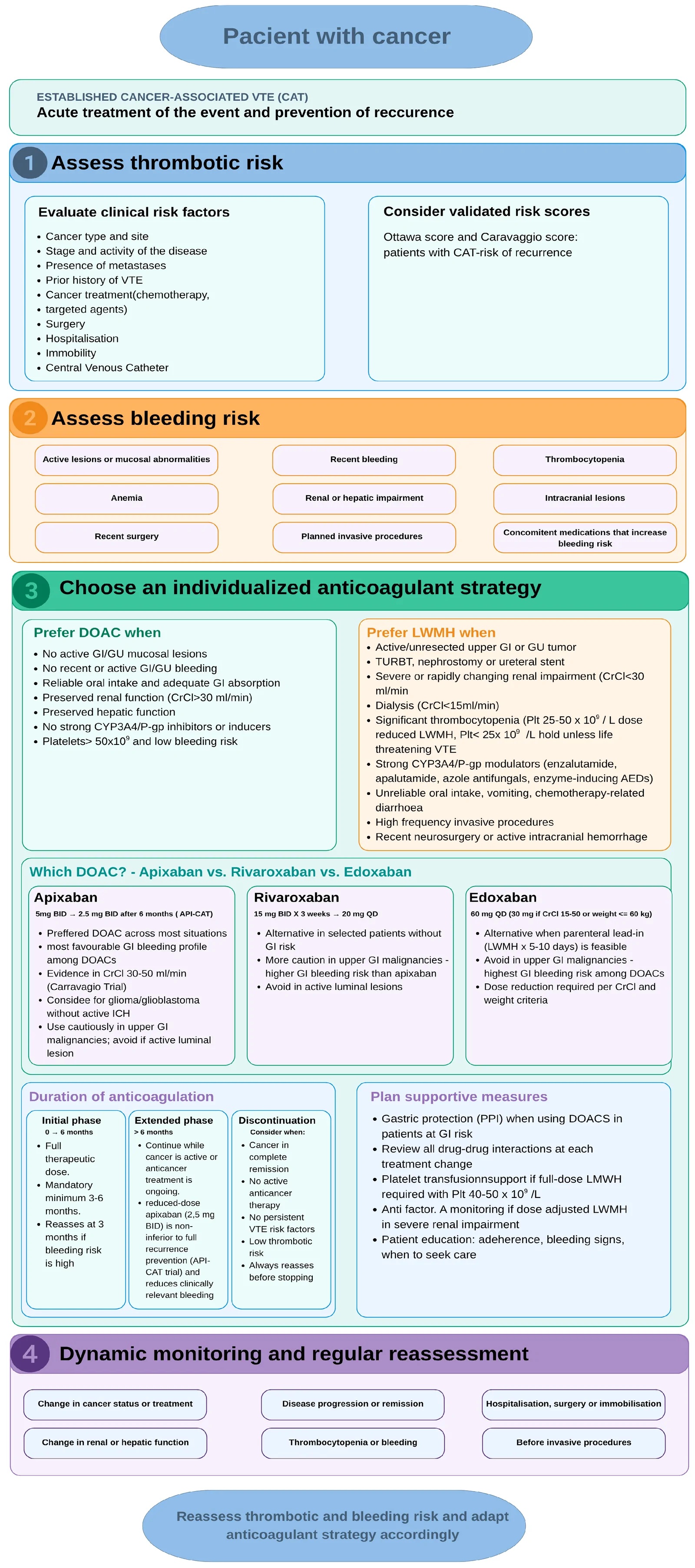
Practical clinical algorithm [14,42,43,44,45,46].

The pragmatic implementation of personalized anticoagulation The practical implementation of personalized anticoagulation requires a flexible approach that integrates the indication for prophylaxis or treatment, thrombotic and bleeding risks, patient- and cancer-related factors, and ongoing reassessment (Figure 3).

Study Limitations.

The main limitations of this review include the heterogeneity of the included studies and the limited availability of high-quality evidence for several specific cancer populations. Although we applied a structured study-selection process and assessed the methodological quality of primary studies using design-specific risk-of-bias tools, we cannot entirely rule out potential selection bias in study inclusion. Differences in study design, patient populations, anticoagulant regimens, and outcome definitions may further limit comparability and generalizability. Given the substantial clinical and methodological heterogeneity of the included evidence, we did not perform a quantitative meta-analysis and instead synthesized the evidence narratively. Consequently, the conclusions rely on a qualitative integration of the available evidence rather than on pooled quantitative estimates, which limits the statistical reproducibility of the findings.

## 5. Conclusions

Cancer-associated thrombosis is a major challenge in oncology, requiring a personalized rather than a universal anticoagulation strategy. Current evidence supports individualized anticoagulant selection based on tumor characteristics, thrombotic and bleeding risks, organ function, concomitant anticancer therapies, and patient-related factors. Among currently available direct oral anticoagulants, apixaban appears to have a favorable efficacy–safety profile based on current evidence, whereas rivaroxaban and edoxaban are still appropriate alternatives in carefully selected patients. Low-molecular-weight heparins still serve a central role in patients with active gastrointestinal or genitourinary mucosal tumors, severe thrombocytopenia, advanced renal impairment, significant drug–drug interactions, or other clinical settings associated with a high bleeding risk. Future advances in validated risk evaluation models, biomarker-guided patient stratification, precision medicine, and novel anticoagulant strategies, including factor XI/XIa inhibitors, are projected to further refine individualized anticoagulant therapy and improve outcomes in patients with cancer-associated thrombosis.

## Figures and Tables

**Table 1:** Pivotal clinical trials of anticoagulation in cancer-associated thrombosis.

Study	Design and Treatment	Population/Sample	Efficacy Outcome	Safety Outcome	Clinical Interpretation
CONKO-011 [17,18].	Rivaroxaban versus LMWH for acute CAT.	247 patients	Recurrent VTE and major or clinically relevant bleeding were secondary outcomes; comparative event rates were not reported in the abstract.		Rivaroxaban modestly reduced patient-reported treatment burden compared with injectable LMWH.
ADAM VTE [11]	Apixaban versus dalteparin.	300 patients.	Recurrent VTE: 3.4% vs. 14.1%.	Major bleeding: 0% vs. 2.1%.	Favorable signal for apixaban; relatively small study.
CARAVAGGIO [12]	Apixaban versus dalteparin.	576 vs. 579 patients.	Recurrent VTE: 5.6% vs. 7.9%.	Major bleeding: 3.8% vs. 4.0%.	Strong evidence supporting apixaban in selected CAT patients.
CASTA-DIVA [10]	Rivaroxaban versus dalteparin.	159 patients.	Detailed numerical efficacy data not available in consulted sources.	Detailed numerical safety data not available in consulted sources.	Adds recent RCT evidence, although detailed data are limited.
CANVAS [13]	DOACs versus LMWH in a pragmatic trial.	Recent pragmatic study.	Detailed numerical efficacy data not available in consulted sources.	Detailed numerical safety data not available in consulted sources.	Supports DOAC use in routine clinical practice.
API CAT [14,16]	Apixaban 2.5 mg versus 5 mg twice daily for 12 months after ≥6 months of prior anticoagulation	1766 patients: 866 vs. 900.	Recurrent VTE: 2.1% vs. 2.8%; reduced-dose apixaban was noninferior.	Clinically relevant bleeding: 12.1% vs. 15.6%.	Reduced-dose apixaban maintained efficacy and reduced clinically relevant bleeding during extended anticoagulation
EVE [14,15,16].	Apixaban 2.5 mg versus 5 mg twice daily for 12 months after 6–12 months of prior anticoagulation.	360 patients: 179 vs. 181.	Recurrent VTE or arterial thrombosis: 5.0% vs. 5.0%.	Major bleeding or CRNMB: 8.9% vs. 12.2%; major bleeding: 2.8% vs. 2.2%.	Reduced-dose apixaban maintained similar efficacy but did not significantly reduce the primary composite bleeding outcome.

Abbreviations: LMWH: low-molecular-weight heparin; VTE: venous thromboembolism; CRNMB: clinically relevant non-major bleeding; CAT: cancer-associated thrombosis; RCT: randomized controlled trial; DOAC: direct oral anticoagulant.

**Table 2:** Clinical trials of FXI/FXIa-targeting monoclonal antibodies in cancer-associated thrombosis.

FXI/FXIa-Targeting Molecule	Study	Clinical Setting/ Objective	Comparator	Status/Key Findings
Abelacimab [19,26]	ASTER (NCT05171049)	Treatment CAT and prevention of recurrent VTE	Apixaban	Terminated in 2026 after an interim efficacy analysis indicated inferior efficacy versus apixaban; complete comparative results have not been published
Abelacimab [19,26]	MAGNOLIA (NCT05171075)	Treatment of CAT in patients with gastrointestinal or genitourinary cancers at increased bleeding risk	Dalteparin	Terminated in 2026 following further strategic review; definitive comparative results have not been reported
REGN7508 [27]	ROXI-CAT-I	Primary thromboprophylaxis in patients with cancer at increased VTE risk	Placebo	Ongoing
REGN7508 [28]	ROXI-CAT-II	Treatment and secondary prevention of VTE in patients with cancer	Apixaban	Ongoing
REGN7508/REGN9933 [19]	ROXI-CATH	Prevention of catheter-related thrombosis in patients with cancer	—	Phase III completed
Gruticibart (xisomab 3G3) [19,29]	Phase II study	Prevention of PICC/CVC-associated thrombosis in patients with cancer	Single-arm	Preliminary evidence of favorable tolerability and potential efficacy

Abbreviations: VTE: venous thromboembolism; CAT: cancer-associated thrombosis; CVC: central venous catheter; PICC: peripherally inserted central catheter.

**Table 3:** Risk-of-bias assessment of the included studies using the Cochrane RoB 2 tool.

Study	Random allocation sequence?	Allocation concealed?	Baseline differences suggest randomization problem?	Participants aware of intervention?	Carers/interventionists aware?	Deviations due to trial context?	Deviations likely to affect outcome?	Deviations balanced between groups?	Appropriate analysis used?	Substantial impact of inappropriate analysis?	Outcome data available for all/nearly all?	Evidence result not biased by missing data?	Missingness related to true outcome?	Relationship likely outcome-dependent?	Outcome measurement inappropriate?	Measurement could differ between groups?	Outcome assessors aware of intervention?	Awareness could influence assessment?	Awareness likely influenced assessment?	Multiple eligible outcome measurements?	Multiple eligible analyses?	Result selected based on results?
McBane et al. 2020 (ADAM VTE) [11]	Y	PY	N	Y	Y	PY	PN	PN	PY	PN	PY	Y	PN	PN	N	N	N	NA	NA	PY *	N *	N *
Agnelli et al. 2020 (CARAVAGGIO) [12]	Y	PY	N	Y	Y	PY	PN	PN	Y	PN	Y	NA	NA	NA	N	N	N	NA	NA	PY *	N *	N *
Planquette et al. 2022 (CASTA-DIVA) [10]	Y	Y	N	Y	Y	PN	NA	NA	Y	NA	PY	PY	PN	PN	N	N	N	NA	NA	PY *	N *	N *
Bertoletti et al. 2022 (ASTER)—terminated; no results published [19,26]	-	-	-	-	-	-	-	-	-	-	-	-	-	-	-	-	-	-	-	-	-	-
Magnolia 2022—terminated; no results published [19,26]	-	-	-	-	-	-	-	-	-	-	-	-	-	-	-	-	-	-	-	-	-	-
Schrag et al. 2023 (CANVAS) [13]	Y	Y	N	Y	Y	PY	Y	N	N	PY	Y	NA	NA	NA	N	N	N	NA	NA	N	PN	N
McBane et al. 2024 (EVE) [14,15,16]	Y	PY	N	N	N	N	NA	NA	PY	PN	PY	PY	PN	PN	N	N	N	NA	NA	PY *	N *	N *
Zwicker et al. 2026 (ROXI-CAT-I) [27]	PY	PY	NI	N	PN	NI	NI	NI	NI	NI	NI	NI	NI	NI	N	N	PN	NA	NA	NI	NI	NI
Zwicker et al. 2026 (ROXI-CATH) [19]	PY	PY	NI	PN	PN	NA	NA	NA	NI	NA	Y	NA	NA	N	N	N	PN	NA	NA	PN	NI	NI
ROXI-CAT-II—Ongoing; no results published [28]	-	-	-	-	-	-	-	-	-	-	-	-	-	-	-	-	-	-	-	-	-	-
Mahé et al. 2025 (API-CAT) [14,16]	Y	Y	N	N	N	N	NA	NA	Y	NA	Y	NA	NA	NA	N	N	N	NA	NA	N	N	N
Sinn et al. 2025 (CONKO-011) [17,18]	Y	PY	N	Y	Y	PY	PY	PY	N	Y	N	PN	PY	PY	N	Y	Y	Y	Y	N	N	N
Wang et al. 2025 (START)—Ongoing; no results published [30]	-	-	-	-	-	-	-	-	-	-	-	-	-	-	-	-	-	-	-	-	-	-

Abbreviations: Y: yes; PY: probably yes; PN: probably no; N: no; NI: no information; NA: not applicable/not assessable; *: provisional assessment pending verification against the prespecified trial protocol, trial registration, and/or statistical analysis plan.

**Table 4:** Summary of domain-level and overall risk-of-bias judgements for randomized studies using the Cochrane RoB 2 tool.

Study	Randomization Process	Deviations from Intended Interventions	Missing Outcome Data	Measurement of the Outcome	Selection of the Reported Result	Overall Risk of Bias
McBane et al. 2020 (ADAM VTE) [11]	Low risk	Low risk	Low risk	Low risk	Low risk *	Low risk *
Agnelli et al. 2020 (CARAVAGGIO) [12]	Low risk	Low risk	Low risk	Low risk	Low risk *	Low risk *
Planquette et al. 2022 (CASTA-DIVA) [10]	Low risk	Low risk	Low risk	Low risk	Low risk *	Low risk *
Bertoletti et al. 2022 (ASTER) [19,26]	Not assessable	Not assessable	Not assessable	Not assessable	Not assessable	Not assessable—terminated, no published results
MAGNOLIA, 2022 [19,26]	Not assessable	Not assessable	Not assessable	Not assessable	Not assessable	Not assessable—terminated, no published results
Schrag et al. 2023 (CANVAS) [13]	Low risk	High risk	Low risk	Low risk	Low risk	High risk
McBane et al. 2024 (EVE) [14,15,16].	Low risk	Low risk	Low risk	Low risk	Low risk *	Low risk *
Zwicker et al. 2026 (ROXI-CAT-I) [27]	Some concerns	Some concerns	Some concerns	Low risk	Some concerns	Some concerns
Zwicker et al. 2026 (ROXI-CATH) [19]	Some concerns	Some concerns	Low risk	Low risk	Some concerns	Some concerns
ROXI-CAT-II [28]	Not assessable	Not assessable	Not assessable	Not assessable	Not assessable	Not assessable—ongoing, no published results
Mahé et al. 2025 (API-CAT) [14,16]	Low risk	Low risk	Low risk	Low risk	Low risk	Low risk
Sinn et al. 2025 (CONKO-011) [17,18].	Low risk	High risk	High risk	High risk	Low risk	High risk
Wang et al. 2025 (START) [30]	Not assessable	Not assessable	Not assessable	Not assessable	Not assessable	Not assessable—protocol/ongoing trial †

Abbreviations: *: provisional assessment pending verification against the prespecified trial protocol, trial registration, and/or statistical analysis plan; †: ongoing trial with no published results; risk-of-bias assessment not assessable.

**Table 5:** Comparative analysis—guidelines for CAT.

Guideline	Key Updates/ New Elements	Preferred Treatment	Duration
ASCO 2023 [41]	Apixaban was added as an option for VTE treatment. Apixaban or rivaroxaban may also be considered for extended postoperative thromboprophylaxis in selected patients.	LMWH, apixaban, rivaroxaban, or edoxaban. VKAs are reserved as an alternative.	At least 6 months; continuation is recommended in patients with active or metastatic cancer or ongoing anticancer treatment.
ESMO 2023 [42]	Consolidates the role of DOACs in CAT.	LMWH or DOACs. Ambulatory thromboprophylaxis is considered only in patients at increased VTE risk identified using validated risk-assessment models.	Individualized according to the clinical context, cancer activity, and thrombotic and bleeding risks.
NCCN 2024/2025 [14,46]	The 2025 update includes the option of reduced-dose apixaban (2.5 mg twice daily) after 6 months of treatment, with particular caution in patients with thrombocytopenia or gastrointestinal surgery.	DOACs are preferred in patients without gastric or gastroesophageal lesions; LMWH is preferred in patients with such lesions or a high bleeding risk.	At least 3 months and generally for as long as cancer remains active or anticancer therapy continues. For non-catheter-related DVT or PE, anticoagulation may be continued indefinitely while cancer is active or thrombotic risk persists.
BSH 2024 [43]	The second edition emphasizes individualized selection between LMWH and DOACs and continuation of anticoagulation in active cancer.	LMWH or DOACs, selected according to bleeding risk, drug–drug interactions, and the clinical context.	Continuation beyond 6 months is recommended in patients with active cancer when the benefit–risk balance remains favorable.
SEOM 2024 [44]	Distinguishes an initial treatment phase (3–6 months) from extended treatment beyond 6 months.	LMWH is preferred in selected patients with GI/GU cancers and high bleeding risk; apixaban may be considered as an alternative.	At least 6 months, followed by individualized continuation with periodic reassessment.
SEMI 2024/2025 [45]	Provides practical recommendations for acute treatment (3–6 months), extended therapy, recurrent VTE during anticoagulation, thrombocytopenia, and catheter-related thrombosis.	LMWH or DOACs in appropriately selected patients, according to individual patient and cancer characteristics.	At least 3–6 months; continuation beyond 6 months is individualized according to cancer activity and the benefit–risk balance.

Abbreviations: ASCO: American Society of Clinical Oncology; ESMO: European Society for Medical Oncology; BSH: British Society for Haematology; SEOM: Spanish Society of Medical Oncology; SEMI: Spanish Society of Internal Medicine; NCCN: National Comprehensive Cancer Network; VTE: venous thromboembolism; LMWH: low-molecular-weight heparin; DOAC: direct oral anticoagulant; GI: gastrointestinal; GU: genitourinary; CAT: cancer-associated thrombosis.

**Table 6:** Suggested anticoagulation strategy for cancer-associated thrombosis according to platelet count in patients with thrombocytopenia.

Platelet Count	Clinical Scenario	Recommended Strategy	Key Considerations
>50 × 10^9^/L [73]	Acute or chronic CAT without active bleeding	Full-dose therapeutic anticoagulation	LMWH or DOAC may be used according to cancer type and bleeding risk. DOACs should be reserved for carefully selected patients.
	Platelet recovery after thrombocytopenia	Resume full-dose anticoagulation	Resume therapeutic-dose LMWH or DOAC once platelet count is stable and no active bleeding is present.
25–50 × 10^9^/L [30,71]	Acute high-risk VTE (proximal PE, extensive proximal DVT recurrent/progressive VTE)	Full-dose LMWH with platelet transfusion support	Maintain platelet count ≥40–50 × 10^9^/L whenever possible. Individualize according to bleeding risk.
	Lower-risk or subacute VTE (distal DVT, catheter-related thrombosis, isolated subsegmental PE, VTE > 30 days)	Reduced-dose or prophylactic-dose LMWH	Consider dose reduction or temporary interruption according to thrombotic and bleeding risk.
<25 × 10^9^/L [71,75]	Most clinical situations	Temporary interruption of anticoagulation	Restart anticoagulation after platelet recovery. In life-threatening VTE, multidisciplinary discussion is recommended.
	Life-threatening acute VTE	Individualized management	Consider platelet transfusion, carefully monitored UFH or LMWH, and alternative supportive strategies.
Active bleeding [71]	Any platelet count	Hold anticoagulation and control the bleeding source	Restart anticoagulation only after hemostasis and reassessment of thrombotic and bleeding risks.

Abbreviations: CAT: cancer-associated thrombosis; LMWH: low-molecular-weight heparin; DOAC: direct oral anticoagulant; VTE: venous thromboembolism; PE: pulmonary embolism; DVT: deep vein thrombosis; UFH: unfractionated heparin.

**Table 7:** Clinical risk assessment models used in cancer-associated thrombosis.

Score	Purpose	Main Variables	Role in CAT
Ottawa [109]	Predict recurrent VTE	Sex, lung cancer, breast cancer, stage I disease, previous VTE	Limited discrimination
Modified Ottawa [109]	Predict recurrent VTE	Modified tumor-stage variables	Modest performance
Caravaggio [105]	Predict recurrent VTE	Symptomatic VTE, pancreatic cancer, metastases, adenocarcinoma, platinum/fluoropyrimidines	Best validated recent model
VTE-BLEED [110]	Predict bleeding	Active cancer, anemia, renal dysfunction, previous bleeding, age, hypertension	Not routinely recommended in CAT
RIETE [111]	Predict bleeding	Cancer, anemia, renal failure, recent bleeding, age	Supportive only

Abbreviations: VTE: venous thromboembolism; CAT: cancer-associated thrombosis; VTE-BLEED: bleeding risk prediction score for patients with venous thromboembolism; RIETE: Computerized Registry of Venous Thromboembolic Disease.

## Data Availability

No new data were created or analyzed in this study. Data sharing is not applicable to this article.

## Abbreviations

The following abbreviations are used in this manuscript:

ADP	Adenosine diphosphate
AI	Artificial intelligence
aPTT	Activated partial thromboplastin time
ASCO	American Society of Clinical Oncology
BSH	British Society for Haematology
CAT	Cancer-associated thrombosis
CRNMB	Clinically relevant non-major bleeding
CrCl	Creatinine clearance
CRT	Catheter-related thrombosis
CVC	Central venous catheter
CYP3A4	Cytochrome P450 3A4
DNA	Deoxyribonucleic acid
DOAC	Direct oral anticoagulant
DVT	Deep vein thrombosis
ERAS	Enhanced Recovery After Surgery
ESMO	European Society for Medical Oncology
FIX	Coagulation factor IX
FXI	Coagulation factor XI
FXIa	Activated coagulation factor XI
FXII	Coagulation factor XII
FXIIa	Activated coagulation factor XII
GI	Gastrointestinal
GU	Genitourinary
HCC	Hepatocellular carcinoma
ICH	Intracranial hemorrhage
INR	International normalized ratio
LMWH	Low-molecular-weight heparin
NCCN	National Comprehensive Cancer Network
NETs	Neutrophil extracellular traps
PARP	Poly(ADP-ribose) polymerase
PE	Pulmonary embolism
P-gp	P-glycoprotein
PICC	Peripherally inserted central catheter
PRISMA	Preferred Reporting Items for Systematic Reviews and Meta-Analyses
RCT	Randomized controlled trial
RIETE	Registro Informatizado de la Enfermedad TromboEmbólica (Computerized Registry of Venous Thromboembolic Disease)
SEMI	Spanish Society of Internal Medicine
SEOM	Spanish Society of Medical Oncology
START	Strategies for Anticoagulation in Patients with Thrombocytopenia and Cancer-Associated Thrombosis
TESEO	Thrombosis and Neoplasia Registry of SEOM
TURBT	Transurethral resection of bladder tumor
UFH	Unfractionated heparin
VEGF	Vascular endothelial growth factor
VEGFR-TKI	Vascular endothelial growth factor receptor tyrosine kinase inhibitor
VKA	Vitamin K antagonist
VTE	Venous thromboembolism
VTE-BLEED	Bleeding-risk prediction score for patients with venous thromboembolism
